# Tris[2-(2*H*-indazol-2-yl)eth­yl]amine

**DOI:** 10.1107/S1600536812022027

**Published:** 2012-05-26

**Authors:** Saúl Ovalle, Perla Elizondo Martínez, Nancy Pérez Rodríguez, Sylvain Bernès, Marcos Flores-Alamo

**Affiliations:** a Laboratorio de Química Industrial, CELAES, Facultad de Ciencias Químicas, UANL, Avenidad Universidad s/n, 66450 San Nicolás de los Garza, NL, Mexico; b DEP Facultad de Ciencias Químicas, UANL, Guerrero y Progreso s/n, Col. Treviño, 64570 Monterrey, NL, Mexico; c Facultad de Química, Universidad Nacional Autónoma de México, México DF 04510, Mexico

## Abstract

The title tertiary amine, C_27_H_27_N_7_, a potential tripodal ligand for coordination chemistry, crystallizes with the central N atom located on a threefold axis of a trigonal cell. The *gauche* conformation of the N(amime)—CH_2_—CH_2_—N(indazole) chain [torsion angle = −64.2 (2)°] places the pendant 2*H*-indazole heterocycles surrounding the symmetry axis, affording a claw-like shaped mol­ecule. Two symmetry-related indazole planes in the mol­ecule make an acute angle of 60.39 (4)°. The lone pair of the tertiary N atom is located inside the cavity, and should thus be inactive (as a ligand). In the crystal, neither significant π–π nor C—H⋯π inter­actions between molecules are found.

## Related literature

For the pharmacological properties of indazoles, see: Cerecetto *et al.* (2005 ▶); Ryu *et al.* (2001 ▶); Teixeira *et al.* (2009 ▶). For isomerism in indazoles, see: Teixeira *et al.* (2006 ▶); Alkorta & Elguero (2005 ▶). For structures of related bis-(2*H*-indazoles), see: Rodríguez de Barbarín *et al.* (2006 ▶); Ovalle *et al.* (2011 ▶). For the structure of the precursor used in the synthesis of the title compound, see: McKee *et al.* (2006 ▶).
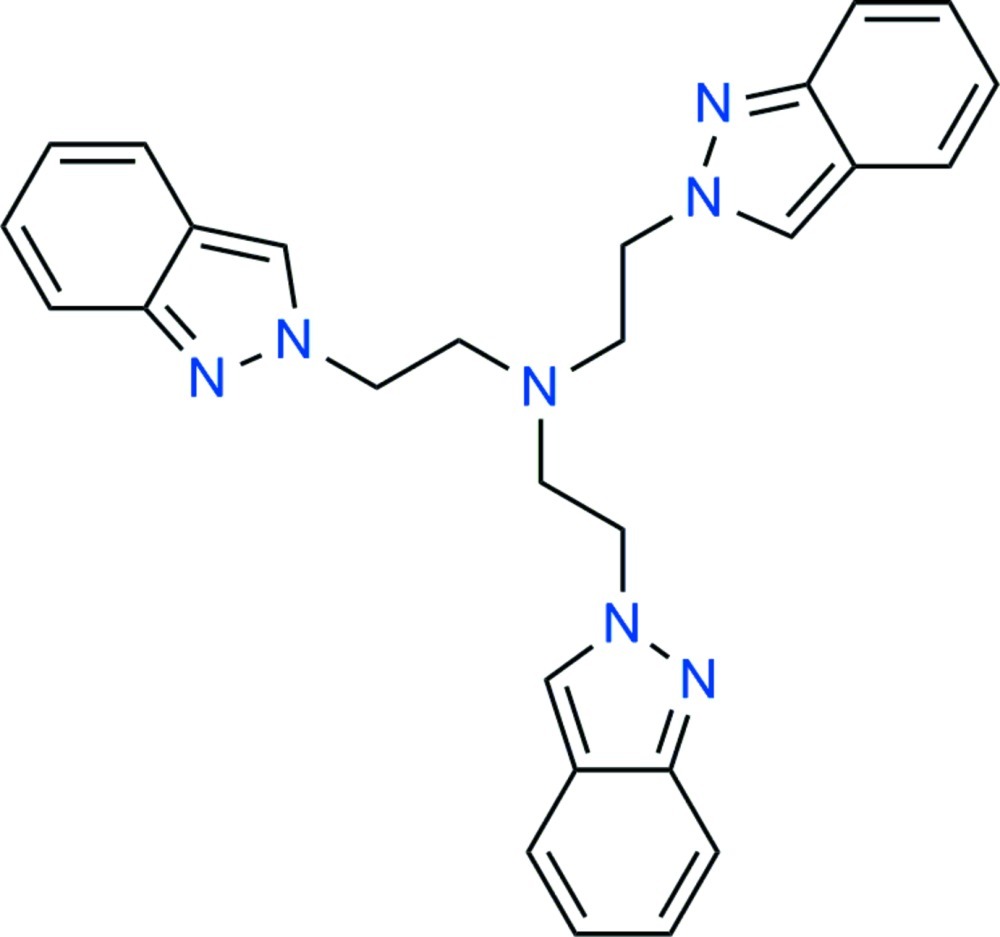


## Experimental

### 

#### Crystal data

C_27_H_27_N_7_
*M* *_r_* = 449.56
Trigonal, 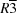

*a* = 13.7314 (15) Å
*c* = 22.235 (3) Å
*V* = 3630.8 (8) Å^3^
*Z* = 6
Mo *K*α radiation
μ = 0.08 mm^−1^
*T* = 130 K
0.40 × 0.20 × 0.20 mm

#### Data collection

Oxford Diffraction Xcalibur Atlas Gemini diffractometer
Absorption correction: multi-scan [*CrysAlis PRO* (Oxford Diffraction, 2009 ▶); based on expressions derived by Clark & Reid (1995 ▶)] *T*_min_ = 0.509, *T*_max_ = 1.000
2900 measured reflections
1404 independent reflections
852 reflections with *I* > 2σ(*I*)
*R*_int_ = 0.035

#### Refinement

*R*[*F* ^2^ > 2σ(*F* ^2^)] = 0.045
*wR*(*F* ^2^) = 0.131
*S* = 0.96
1404 reflections
103 parameters
H-atom parameters constrained
Δρ_max_ = 0.12 e Å^−3^
Δρ_min_ = −0.15 e Å^−3^

### 

Data collection: *CrysAlis CCD* (Oxford Diffraction, 2009 ▶); cell refinement: *CrysAlis RED* (Oxford Diffraction, 2009 ▶); data reduction: *CrysAlis RED*; program(s) used to solve structure: *SHELXS97* (Sheldrick, 2008 ▶); program(s) used to refine structure: *SHELXL97* (Sheldrick, 2008 ▶); molecular graphics: *SHELXTL* (Sheldrick, 2008 ▶); software used to prepare material for publication: *SHELXTL*.

## Supplementary Material

Crystal structure: contains datablock(s) I, global. DOI: 10.1107/S1600536812022027/lr2063sup1.cif

Structure factors: contains datablock(s) I. DOI: 10.1107/S1600536812022027/lr2063Isup2.hkl

Supplementary material file. DOI: 10.1107/S1600536812022027/lr2063Isup3.cml

Additional supplementary materials:  crystallographic information; 3D view; checkCIF report

## Figures and Tables

**Fig. 1.**
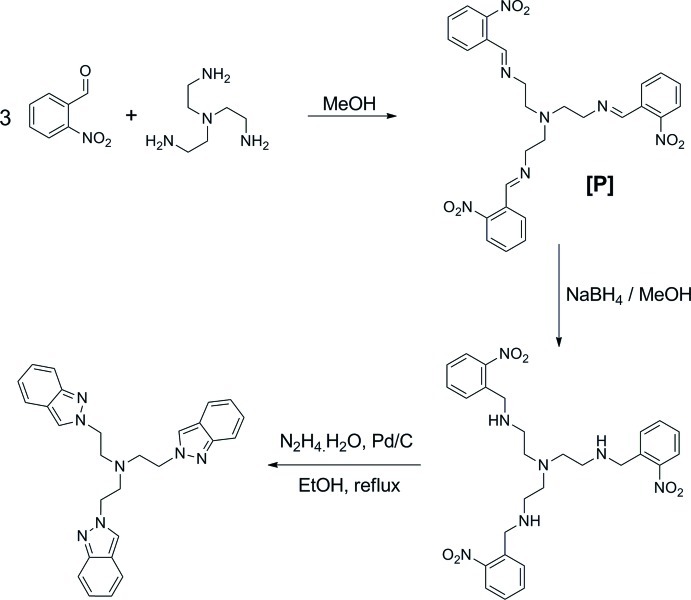
Synthetic route for the title compound. The X-ray structure of the precursor [P] has been reported (McKee et al., 2006).

**Fig. 2.**
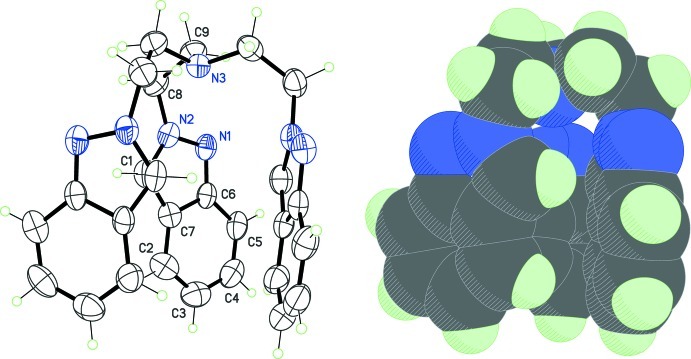
ORTEP-like view of the title molecule, with displacement ellipsoids at the 30% probability level for non-H atoms. Unlabeled atoms are generated by symmetry codes: -x + y, 1 - x, z and 1 - y, 1 + x-y, z. The figure on the right is a space filling representation in the same orientation, showing the full shape of the molecule.

## supplementary crystallographic information

### Comment

The interest in obtaining the title molecule, a new 2*H*-indazole derivative, is due to the potential applications for this class of compounds. Regarding pharmacological activity, these include anti-inflammatory, antitumor and antidepressants drugs (Cerecetto *et al.*, 2005). These molecules have also been used in agriculture as selective herbicides (Ryu *et al.*, 2001) and as precursors of other compounds to increase their biological activity and specificity (Teixeira *et al.*, 2009). The chemistry of 2*H*-indazole remains less studied compared to its tautomer 1*H*-indazole, in part because the latter is thermodynamically more stable for the majority of derivatives (Teixeira *et al.*, 2006). However the opposite situation also occurs in some cases (Alkorta & Elguero, 2005).

The title molecule is a tris-(2*H*-indazole) compound derived from a tertiary amine (Fig. 1). The molecule is placed on a threefold axis in space group *R*3 (*Z*' = 1/3). Each arm contains a *gauche* N_amime_—CH_2_—CH_2_—N_indazole_ chain [torsion angle: -64.2 (2)°], forming a claw-like geometry (Fig. 2). The geometry for the tertiary N atom (N3) is consistent with the presence of the lone pair inside the molecular cavity. The three symmetry-related indazole heterocycles in the molecule are arranged in such a way that no strong interactions are present. The C1—H1A group interacts weakly with the pyrazole ring of the following arm: the separation H1A···*Cg^i^* is 2.85 Å and the angle C1—H1A···*Cg^i^* is 128° (*Cg* is the centroid of ring N1/N2/C1/C7/C6 and *i* stands for symmetry code: 1 - *y*, 1 + *x*-*y*, *z*). The angle between two indazole planes in the molecule is 60.39 (4)°. Other geometric parameters compare well with those previously reported for bis-(2*H*-indazole) compounds (Rodríguez de Barbarín *et al.*, 2006; Ovalle *et al.*, 2011).

### Experimental

The title molecule was obtained by a three steps reaction (Fig. 1). Condensation between tris(2-aminoethyl)amine and 2-nitrobenzaldehyde produced the corresponding tris-imine (McKee *et al.*, 2006). Selective reduction of imine bonds with sodium borohydride in methanol gave the corresponding amine, which was isolated. Then, 0.046 g of Pd/C was added to a solution of this intermediate (0.005 mol) in ethanol. The mixture was refluxed for 4 h, with addition of hydrazine monohydrate (0.110 mol) during the first 3 h. The resulting mixture was filtered, distilled, and the organic phase was extracted. The product was purified by column chromatography with silica gel and methanol as eluent. Suitable crystals were obtained by slow evaporation of an ethanol solution at 298 K. *M*.p. 445 K; analysis found (calc. for C_27_H_27_N_7_): C 71.46 (72.14), H 5.98 (6.05), N 22.56% (21.81%); IR RTA: 3119 (CH Ar. ν_s_), 2953 (–CH_2_– ν_s_), 1623 (C=N Ar. δ_s_), 1471, 1512 (C=C Ar. ν_s_ and ν_as_). ^1^H NMR (300 MHz, CDCl_3_): 7.65 (d, 3*H*, ArH), 7.29 (t, 3*H*, ArH), 7.05 (t, 3*H*, ArH), 7.00 (d, 3*H*, ArH), 5.58 (s, 3*H*, ArH), 4.00 (t, 6*H*, CH_2_), 3.09 (t, 6*H*, CH_2_) p.p.m.. ^13^C NMR: 150–115 (Ar), 55–50 (–CH_2_–).

### Refinement

All H atoms were placed in idealized positions and refined as riding to their parent C atoms, with bond lengths fixed to 0.97 (methylene CH_2_) or 0.93 Å (aromatic CH). Isotropic displacement parameters for H atoms were calculated as *U*_iso_(H) = 1.2 *U*_eq_(carrier atom).

### Crystal data

**Table:**

C_27_H_27_N_7_	*D*_x_ = 1.234 Mg m^−^^3^
*M**_r_* = 449.56	Melting point: 445 K
Trigonal, *R*3	Mo *K*α radiation, λ = 0.71073 Å
Hall symbol: -R 3	Cell parameters from 1214 reflections
*a* = 13.7314 (15) Å	θ = 3.5–29.5°
*c* = 22.235 (3) Å	µ = 0.08 mm^−^^1^
*V* = 3630.8 (8) Å^3^	*T* = 130 K
*Z* = 6	Plate, orange
*F*(000) = 1428	0.40 × 0.20 × 0.20 mm

### Data collection

**Table:**

Oxford Diffraction Xcalibur Atlas Gemini diffractometer	1404 independent reflections
Radiation source: Enhance (Mo) X-ray Source	852 reflections with *I* > 2σ(*I*)
Graphite monochromator	*R*_int_ = 0.035
Detector resolution: 10.4685 pixels mm^-1^	θ_max_ = 25.3°, θ_min_ = 3.6°
ω scans	*h* = −16→11
Absorption correction: multi-scan [*CrysAlis PRO* (Oxford Diffraction, 2009); based on expressions derived by Clark & Reid (1995)]	*k* = −11→16
*T*_min_ = 0.509, *T*_max_ = 1.000	*l* = −18→26
2900 measured reflections	

### Refinement

**Table:**

Refinement on *F*^2^	Primary atom site location: structure-invariant direct methods
Least-squares matrix: full	Secondary atom site location: difference Fourier map
*R*[*F*^2^ > 2σ(*F*^2^)] = 0.045	Hydrogen site location: inferred from neighbouring sites
*wR*(*F*^2^) = 0.131	H-atom parameters constrained
*S* = 0.96	*w* = 1/[σ^2^(*F*_o_^2^) + (0.0792*P*)^2^] where *P* = (*F*_o_^2^ + 2*F*_c_^2^)/3
1404 reflections	(Δ/σ)_max_ < 0.001
103 parameters	Δρ_max_ = 0.12 e Å^−^^3^
0 restraints	Δρ_min_ = −0.15 e Å^−^^3^
0 constraints	

### Fractional atomic coordinates and isotropic or equivalent isotropic displacement parameters (Å^2^)

**Table:**

	*x*	*y*	*z*	*U*_iso_*/*U*_eq_	
N1	0.06603 (12)	0.53619 (12)	0.91972 (8)	0.0708 (5)	
N2	0.14166 (12)	0.50358 (11)	0.93250 (7)	0.0667 (5)	
N3	0.3333	0.6667	1.01076 (10)	0.0624 (7)	
C1	0.19002 (14)	0.48969 (15)	0.88423 (10)	0.0724 (6)	
H1A	0.2435	0.4671	0.8839	0.087*	
C2	0.16242 (17)	0.51736 (19)	0.77209 (10)	0.0881 (7)	
H2A	0.2129	0.4981	0.7558	0.106*	
C3	0.1031 (2)	0.54814 (19)	0.73614 (11)	0.0956 (8)	
H3A	0.1128	0.5501	0.6947	0.115*	
C4	0.0267 (2)	0.57740 (17)	0.76090 (12)	0.0932 (8)	
H4A	−0.0127	0.5990	0.7353	0.112*	
C5	0.00892 (17)	0.57496 (15)	0.82125 (12)	0.0822 (6)	
H5A	−0.0423	0.5938	0.8369	0.099*	
C6	0.06970 (13)	0.54348 (13)	0.85902 (10)	0.0625 (5)	
C7	0.14644 (13)	0.51492 (14)	0.83448 (9)	0.0647 (5)	
C8	0.15877 (17)	0.48420 (16)	0.99499 (9)	0.0802 (6)	
H8A	0.2021	0.4460	0.9963	0.096*	
H8B	0.0863	0.4351	1.0133	0.096*	
C9	0.21862 (15)	0.59058 (16)	1.03092 (8)	0.0771 (6)	
H9A	0.1761	0.6295	1.0287	0.093*	
H9B	0.2206	0.5714	1.0727	0.093*	

### Atomic displacement parameters (Å^2^)

**Table:**

	*U*^11^	*U*^22^	*U*^33^	*U*^12^	*U*^13^	*U*^23^
N1	0.0597 (10)	0.0648 (9)	0.0921 (13)	0.0343 (8)	0.0033 (8)	0.0052 (8)
N2	0.0544 (9)	0.0587 (9)	0.0845 (12)	0.0265 (7)	−0.0009 (8)	0.0072 (7)
N3	0.0626 (10)	0.0626 (10)	0.0621 (16)	0.0313 (5)	0.000	0.000
C1	0.0472 (10)	0.0709 (12)	0.0993 (16)	0.0298 (9)	0.0048 (10)	0.0007 (10)
C2	0.0654 (13)	0.0928 (16)	0.0912 (17)	0.0283 (11)	−0.0011 (11)	−0.0138 (12)
C3	0.0872 (16)	0.0865 (16)	0.0829 (17)	0.0209 (13)	−0.0145 (13)	−0.0056 (12)
C4	0.0903 (16)	0.0622 (13)	0.110 (2)	0.0255 (12)	−0.0399 (14)	−0.0024 (12)
C5	0.0740 (13)	0.0594 (12)	0.1138 (19)	0.0338 (10)	−0.0214 (12)	−0.0076 (11)
C6	0.0464 (10)	0.0429 (9)	0.0907 (15)	0.0168 (8)	−0.0048 (9)	0.0011 (8)
C7	0.0417 (9)	0.0580 (11)	0.0835 (14)	0.0168 (8)	−0.0031 (9)	−0.0060 (9)
C8	0.0712 (13)	0.0675 (13)	0.0906 (15)	0.0262 (10)	−0.0005 (10)	0.0155 (10)
C9	0.0736 (13)	0.0812 (14)	0.0715 (14)	0.0349 (11)	0.0126 (10)	0.0132 (10)

### Geometric parameters (Å, º)

**Table:**

N1—N2	1.351 (2)	C3—C4	1.410 (3)
N1—C6	1.353 (2)	C3—H3A	0.9300
N2—C1	1.325 (2)	C4—C5	1.361 (3)
N2—C8	1.456 (2)	C4—H4A	0.9300
N3—C9^i^	1.4588 (19)	C5—C6	1.396 (3)
N3—C9	1.4588 (19)	C5—H5A	0.9300
N3—C9^ii^	1.4588 (19)	C6—C7	1.405 (2)
C1—C7	1.382 (3)	C8—C9	1.499 (3)
C1—H1A	0.9300	C8—H8A	0.9700
C2—C3	1.351 (3)	C8—H8B	0.9700
C2—C7	1.402 (3)	C9—H9A	0.9700
C2—H2A	0.9300	C9—H9B	0.9700
			
N2—N1—C6	103.18 (13)	C4—C5—H5A	120.9
C1—N2—N1	113.64 (15)	C6—C5—H5A	120.9
C1—N2—C8	127.49 (17)	N1—C6—C5	128.11 (18)
N1—N2—C8	118.84 (15)	N1—C6—C7	111.85 (16)
C9^i^—N3—C9	110.99 (10)	C5—C6—C7	120.0 (2)
C9^i^—N3—C9^ii^	110.99 (11)	C1—C7—C2	135.71 (19)
C9—N3—C9^ii^	110.99 (11)	C1—C7—C6	103.79 (18)
N2—C1—C7	107.54 (17)	C2—C7—C6	120.51 (18)
N2—C1—H1A	126.2	N2—C8—C9	112.99 (15)
C7—C1—H1A	126.2	N2—C8—H8A	109.0
C3—C2—C7	118.8 (2)	C9—C8—H8A	109.0
C3—C2—H2A	120.6	N2—C8—H8B	109.0
C7—C2—H2A	120.6	C9—C8—H8B	109.0
C2—C3—C4	120.6 (2)	H8A—C8—H8B	107.8
C2—C3—H3A	119.7	N3—C9—C8	113.80 (16)
C4—C3—H3A	119.7	N3—C9—H9A	108.8
C5—C4—C3	121.9 (2)	C8—C9—H9A	108.8
C5—C4—H4A	119.1	N3—C9—H9B	108.8
C3—C4—H4A	119.1	C8—C9—H9B	108.8
C4—C5—C6	118.2 (2)	H9A—C9—H9B	107.7
			
C6—N1—N2—C1	−0.53 (18)	N2—C1—C7—C6	−0.23 (18)
C6—N1—N2—C8	−178.56 (14)	C3—C2—C7—C1	−179.5 (2)
N1—N2—C1—C7	0.49 (19)	C3—C2—C7—C6	0.3 (3)
C8—N2—C1—C7	178.32 (15)	N1—C6—C7—C1	−0.10 (18)
C7—C2—C3—C4	0.1 (3)	C5—C6—C7—C1	179.66 (15)
C2—C3—C4—C5	−0.6 (3)	N1—C6—C7—C2	−179.97 (14)
C3—C4—C5—C6	0.7 (3)	C5—C6—C7—C2	−0.2 (2)
N2—N1—C6—C5	−179.36 (16)	C1—N2—C8—C9	111.2 (2)
N2—N1—C6—C7	0.37 (18)	N1—N2—C8—C9	−71.1 (2)
C4—C5—C6—N1	179.45 (17)	C9^i^—N3—C9—C8	159.04 (17)
C4—C5—C6—C7	−0.3 (2)	C9^ii^—N3—C9—C8	−77.0 (3)
N2—C1—C7—C2	179.62 (19)	N2—C8—C9—N3	−64.2 (2)

Symmetry codes: (i) −*x*+*y*, −*x*+1, *z*; (ii) −*y*+1, *x*−*y*+1, *z*.
